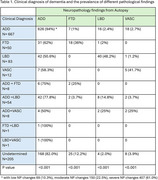# Pathology‐Validated Accuracy of Dementia Diagnosis with Neuropsychiatric Symptoms

**DOI:** 10.1002/alz70857_102059

**Published:** 2025-12-25

**Authors:** Matin Soeizi, Tovia Jacobs, Zafarullah M Chaudhary, Ricardo Osorio, Sakina Ouedraogo Tall

**Affiliations:** ^1^ NYU Grossman School of Medicine, New York, NY, USA; ^2^ New York City Health and Hospitals, New York, NY, USA

## Abstract

**Background:**

Neuropsychiatric symptoms (NPS) of dementia negatively impact patients’ and caregivers' quality of life. These symptoms can complicate clinical assessment and the care of patients living with dementia. High NPS burdens, measured by the Neuropsychiatric Inventory Questionnaire (NPI‐Q), are found to be associated with Lewy Body Dementia (LBD) and Alzheimer Disease Dementia (ADD). Our study compares clinical diagnoses to pathology findings to evaluate diagnostic accuracy in the presence of NPS.

**Method:**

This retrospective longitudinal cohort study utilized participant data from the National Alzheimer's Coordinating Center (NACC). We included participants with recorded delusions, hallucinations, and/or agitation assessed by the NPI‐Q at Mild Cognitive Impairment (MCI) or mild dementia stage. Participants with schizophrenia and unspecified psychiatric disorders were excluded. We studied clinical diagnosis and investigated neuropathology concordance. All statistical analyses were conducted using R software. We used one‐way analysis of variance (ANOVA) for assessment.

**Result:**

Total of 1,089 participants were included, 44.4% were female, race predominantly white (93.7%), average age at the first visit with NPS present was 77.96 years old. The NPI‐Q showed 880 participants had agitation, 296 delusions, and 184 hallucinations. Amongst 667 participants with a clinical diagnosis of ADD, 94% were found to have ADD as main pathology. Of 50 patients with a clinical diagnosis of Frontotemporal Dementia (FTD), only 36% had a concordance finding on NP and 62% were found to have changes consistent with ADD. For the 83 participants with clinical diagnosis of LBD, 48.2% had NP confirming LBD, and 50.6% ADD. Vascular impairment was the clinical diagnosis associated with 12 participants, out of which 41.7% were found to have vascular dementia on NP and the remaining ADD.

**Conclusion:**

Our study revealed that NPS are most frequently associated with ADD pathology. In more than 50% of cases, NPS were inaccurately attributed to another clinical diagnosis in presence of pathology confirmed LBD, FTD, or vascular dementia. More studies are needed to further assist in diagnosis accuracy in cases of dementia with behavioral disturbances.